# Retracted: *VEGF* Genetic Polymorphisms May Contribute to the Risk of Diabetic Nephropathy in Patients with Diabetes Mellitus: A Meta-Analysis

**DOI:** 10.1155/2020/6482307

**Published:** 2020-08-01

**Authors:** The Scientific World Journal

*The Scientific World Journal* has retracted the article titled “*VEGF* Genetic Polymorphisms May Contribute to the Risk of Diabetic Nephropathy in Patients with Diabetes Mellitus: A Meta-Analysis” [1]. This article is one of a series of very similar meta-analyses written by different authors that were published in 2014 and 2015, characterized by searching the complementary and alternative medicine database CISCOM despite the topic not being about CAM [2], as is also the case with this article. The overlaps of wording with these articles are concentrated in the Materials and Methods and Results sections, and a paragraph in the Discussion. The article also mentions “Begger's test,” which is a mistaken combination of “Begg's test” and “Egger's test” and is also characteristic of this series of articles. The article inappropriately mentions other cancers and genes, including gastric cancer in the abstract and “breast cancer” and “IFN-*γ* gene” in Figure 1 in the eligibility exclusion box, whereas the article is about diabetic nephropathy (DN) and *VEGF*.

Additionally, the assessment of publication bias may be incorrect. The statement that “Egger's test also did not display strong statistical evidence for publication bias (allele mode: *t* = 2.92, *P*=0.011 and dominant model: *t* = 2.53, *P*=0.024, resp.)” is made despite the test result being statistically significant and the funnel plots are said to be symmetrical, though the smaller studies (with greater standard error) show greater associations with DN, which is an indication of publication bias.

The authors said they attended a course on writing meta-analyses that lead to incorrect data analysis and they apologised for the mistakes. However, they asked to retract the article (though they did not approve the content of this notice) and this was approved by the editorial board.
